# Using Sequence Similarity Based on CKSNP Features and a Graph Neural Network Model to Identify miRNA–Disease Associations

**DOI:** 10.3390/genes13101759

**Published:** 2022-09-28

**Authors:** Mingxin Li, Yu Fan, Yiting Zhang, Zhibin Lv

**Affiliations:** 1 College of Biomedical Engineering, Sichuan University, Chengdu 610065, China; 2 College of Biology, Southwest Jiaotong University, Chengdu 611756, China; 3 College of Biology, Georgia State University, Atlanta, GA 30302-3965, USA

**Keywords:** miRNA, miRNA sequence similarity, graph neural network, graph auto-encoder

## Abstract

Among many machine learning models for analyzing the relationship between miRNAs and diseases, the prediction results are optimized by establishing different machine learning models, and less attention is paid to the feature information contained in the miRNA sequence itself. This study focused on the impact of the different feature information of miRNA sequences on the relationship between miRNA and disease. It was found that when the graph neural network used was the same and the miRNA features based on the K-spacer nucleic acid pair composition (CKSNAP) feature were adopted, a better graph neural network prediction model of miRNA–disease relationship could be built (AUC = 93.71%), which was 0.15% greater than the best model in the literature based on the same benchmark dataset. The optimized model was also used to predict miRNAs related to lung tumors, esophageal tumors, and kidney tumors, and 47, 47, and 37 of the top 50 miRNAs related to three diseases predicted separately by the model were consistent with descriptions in the wet experiment validation database (dbDEMC).

## 1. Introduction

According to molecular biology, the entire human genome can be divided into regulatory, coding, and noncoding genes. The coding genes are the main carriers of human genetic information, accounting for only 1.5% of the whole human genome [1,2,3]. Previous studies have regarded non-coding genes as transcriptional noise in the coding process. However, microarray experiments have found that non-coding genes can participate in cell activities by interacting with proteins and DNA, affecting gene activation and silencing; RNA splicing, modification, and editing; and protein translation, thus affecting various physiological processes. MicroRNA (miRNA) is a type of non-coding, single-stranded RNA encoded by endogenous genes. The most common role of miRNA is to directly regulate target genes by affecting post-transcriptional gene regulation of promoters. Abnormal expression of miRNA can cause many human diseases, and miRNA can be used as a drug target for disease treatment [4,5]. Therefore, the regulatory role of miRNA in disease expression is significant for a variety of complex human physiological processes and disease pathophysiology. However, traditional experimental methods are often limited. To this end, it is of great significance to speed up the verification process, reduce bias in biological experiments, and establish a method to predict the possible associations quickly and effectively between miRNAs and disease [6].

Common biological experimental methods from the 1990s to the beginning of the 21st century include reverse transcription-polymerase chain reaction (RT-PCR) [7], Northern blotting [8], and microarray profiling [9]. Although these traditional methods can accurately detect correlations between miRNA and disease, they have their limitations. In recent years, a more classical class of algorithm has been developed based on the assumption of similarity measures [10]. This kind of algorithm is based on the assumption that miRNAs with similar functions are also related to similar disease phenotypes and can be sequenced accordingly [11]. Jiang et al. [12] constructed an miRNA association network using probability of interaction by target accessibility (PITA), and they proposed a hypergeometric distribution prediction method based on the human miRNA network. Liu et al. [13] proposed an miRNA–disease association prediction method by random walk on a heterogeneous network constructed by integrating multiple data sources. Zeng et al. [14] applied a link prediction algorithm named the structural perturbation method (SPM) on the miRNA–disease bilayer network to predict potential miRNA–disease associations. Zhang et al. [15] proposed a method using the correlation spectrum and the interaction network between miRNA and target genes to calculate the miRNA–disease association score by using fast linear neighborhood similarity-based network link inference. Mørk et al. [16] proposed a model to calculate the similarity between miRNA and disease based on the distance between target genes and disease genes in the PPI network. Another common method for predicting the miRNA–disease association is based on machine-inferred code similarity (MISIM) proposed by Wang et al. [17] Chen et al. [18] proposed a model called random forest for miRNA–disease association (RFMDA), which integrates disease semantic similarity, disease Gaussian interaction profile kernel similarity, miRNA Gaussian interaction profile kernel similarity, and miRNA functional similarity based on MISIM to predict miRNA–disease association. Zeng et al. [19] developed a neural network model to predict miRNA–disease associations (NNMDA). NNMDA not only aggregated the neighbor information during the process, but also preserved the topology of the original network at the same time. Zhou et al. [20] used similar methods to obtain miRNA and disease similarity networks and used the gradient boosting decision tree (GBDT) algorithm to extract more representative features. Additionally, with the recent great progress of graph neural networks in processing graph data, prediction methods based on graph neural networks have made breakthroughs, and the association between miRNA and disease is more suited to using graph neural networks than other data structures.

A key step in the data preprocessing for all models is to calculate the similarity of miRNAs. Specifically, these methods can be divided into three types: similarity measure-based methods, MISIM-based methods, and miRNA sequence similarity-based methods. Similarity measure-based methods construct an miRNA association network using target gene associations. However, this depends on the association between miRNA and target genes, resulting in high false positive and false negative rates [12,15,16,21,22,23,24,25,26,27,28]. The MISIM-based methods construct an miRNA association network using a disease semantic similarity network [17]. It can be said that related miRNAs have related diseases, but not all miRNAs related to similar diseases are necessarily related [18,20,28,29,30]. The miRNA similarity networks of such methods have the disadvantage of being dependent on known disease similarity networks, and this error is more pronounced for miRNAs that are associated with fewer diseases. The sequence similarity of miRNA is also particularly important for similarity networks. Previous studies have proposed enriching the miRNA similarity networks using miRNA sequence information. Ji et al. [31] integrated various kinds of information to construct a heterogeneous network centered on miRNA and disease, and embedded K-mer sequence features of miRNA into this network. Ji et al. [32] proposed a method using disease semantic similarity and miRNA sequence similarity to construct an miRNA–disease association network; miRNA sequence similarity-based methods effectively quantify the miRNA similarity, solving the problem that miRNA sequences cannot be directly compared due to their different lengths. In conclusion, according to current research, there is still room for improvement in the accuracy and effectiveness of identifying and predicting potential associations between miRNA and disease. Therefore, we enhanced the effective associations between miRNA and disease by using miRNA sequence information, benefitting from the advantage of graph neural networks in finding miRNA–disease associations to construct a feature extraction method based on miRNA sequence similarity information.

In this study, we propose a method to calculate the sequence characteristics and sequence similarity of miRNA using five distinct miRNA sequence characteristics. Then, by integrating disease semantic similarity, miRNA Gaussian interaction profile kernel similarity, and disease Gaussian interaction profile kernel similarity, we construct a new bipartite graph of miRNA and disease. Then, the auto-encoder of a graph neural network is used to predict miRNA–disease association. Moreover, we evaluated prediction performance using 5-fold cross-validation. The model, using sequence similarity based on the composition of k-spaced nucleic acid pair (CKSNAP) features and a graph neural network, achieved an average area under the curve (AUC) of 93.71 ± 0.42%, with an accuracy of 83.69 ± 1.42%, precision of 77.73 ± 2.29%, recall of 94.62 ± 0.97%, and F1-score of 85.31 ± 1.00%. To further verify the performance of this model, case studies on lung, esophageal, and kidney neoplasms were conducted. Respectively, the results showed that 47, 47, and 37 of the top 50 predicted miRNAs for these neoplasms can be confirmed by the database of Differentially Expressed MiRNAs in human Cancers (dbDEMC) [33]. In conclusion, it can be inferred that our model is effective and accurate in identifying potential associations between miRNA and disease.

## 2. Materials and Methods

### 2.1. Human miRNA–Disease Associations

In this study, we adopted the Human microRNA Disease Database (HMDD; v3.2) as the benchmark dataset and directly downloaded the experimentally verified miRNA–disease associations from https://www.cuilab.cn/hmdd (accessed on 1 September 2021) [34]. There are 16,427 high-quality miRNA–disease associations recorded in the HMDD database, including 877 diseases and 901 miRNAs. We adopted the adjacency matrix A to quantify associations between these miRNAs and diseases. If a disease is associated with an miRNA, the value of the element at the corresponding position of matrix A is set to 1, and otherwise to 0.

### 2.2. miRNA Sequence Similarity

In this study, the attribute features of miRNAs were represented by sequence similarity information. We downloaded miRNA sequence information from https://mirbase.org/ftp.shtml and utilized the K-mer (k = 3), Moran, Geary, NMBroto, and CKSNAP (k = 5) methods to obtain the sequence features of miRNAs.

K-mer: We set up a sliding window with a size of 3 and a sliding distance of 1 to obtain occurrence frequencies for all 3-monomere units. Then, each miRNA sequence was converted into a 64-dimensional vector based on the 64 3-monomer combinations. On this basis, we used cosine similarity, euclidean distance, and the Pearson correlation coefficient to calculate miRNA sequence similarity, defined as follows:

(1) $$Kmer\_\cos\_sim\left(i,\;j\right)=\frac{\sum_{j=1}^{n}\sum_{i=1}^{n}\frac{N\left(i\right)}{N}\times \frac{N\left(j\right)}{N}}{\sqrt{\sum_{i=1}^{n}{\left(\frac{N\left(i\right)}{N}\right)}^{2}}\times \sqrt{\sum_{j=1}^{n}{\left(\frac{N\left(j\right)}{N}\right)}^{2}}}\;$$

(2) $$Kmer\_euc\_dist\left(i,j\right)=\sqrt{\overset{n}{\underset{\begin{array}{c} i=1\\ j=1 \end{array}}{\sum }}{\left(\frac{N\left(i\right)}{N}-\frac{N\left(j\right)}{N}\right)}^{2}}\;$$

(3) $$\begin{array}{c} Kmer\_pearson\left(i,j\right)\\ =\frac{N\sum \frac{N\left(i\right)}{N}\times \frac{N\left(j\right)}{N}-\sum \frac{N\left(i\right)}{N}\sum \frac{N\left(j\right)}{N}}{\sqrt{N\sum {\left(\frac{N\left(i\right)}{N}\right)}^{2}-{\left(\sum \frac{N\left(i\right)}{N}\right)}^{2}}\sqrt{N\sum {\left(\frac{N\left(j\right)}{N}\right)}^{2}-{\left(\sum \frac{N\left(j\right)}{N}\right)}^{2}}} \end{array}$$

where *i* represents a nucleotide combination with a length of three, such as AAA, AAC, and AAG; *N(i)* is the number of nucleotide combinations and *N* is the length of a nucleotide sequence.

Moran: Moran describes miRNA by physicochemical parameters of nucleotides. The physical and chemical properties of miRNA include Rise (RNA), Roll(RNA), Shift(RNA), Slide(RNA), Tilt(RNA), Twist(RNA), Entropy(RNA), Adenine content, Purine(AG) content, Hydrophilicity(RNA), Enthalpy(RNA)1, GC content, Entropy(RNA)1, Hydrophilicity(RNA)1, Free energy(RNA), Keto(GT) content, Free energy(RNA)1, Enthalpy(RNA), Stacking energy(RNA), Guanine content, Cytosine content, and Thymine content. On this basis, we calculated the miRNA sequence similarity, defined as follows:

(4) $$Moran\_\cos\_sim\left(i,\;j\right)=\frac{\sum_{j=1}^{n}\sum_{i=1}^{n}M_{i}\times M_{j}}{\sqrt{\sum_{i=1}^{n}{\left(M_{i}\right)}^{2}}\times \sqrt{\sum_{j=1}^{n}{\left(M_{j}\right)}^{2}}}$$

(5) $$Moran\_euc\_dist\left(i,j\right)=\sqrt{\overset{n}{\underset{\begin{array}{c} i=1\\ j=1 \end{array}}{\sum }}{\left(M_{i}-M_{j}\right)}^{2}}$$

(6) $$Moran\_pearson\left(i,j\right)=\frac{N\sum M_{i}\times M_{j}-\sum M_{i}\sum M_{j}}{\sqrt{N\sum M_{i}{}^{2}-{\left(\sum M_{i}\right)}^{2}}\sqrt{N\sum M_{j}{}^{2}-{\left(\sum M_{j}\right)}^{2}}}\;$$

where the Moran feature of miRNA are defined as follows:

(7) $$M\left(d\right)=\frac{\frac{1}{N-d}\sum_{i=1}^{N-d}\left(M_{i}-\bar{M^{\prime }}\right)\left(M_{i+d}-\bar{M\prime }\right)}{\frac{1}{N}\sum_{i=1}^{N}{\left(M_{i}-\bar{M\prime }\right)}^{2}},\;d=1,2,3,\ldots ,\;nlag$$

(8) $$M=\frac{\sum_{r=1}^{len}\frac{M_{r}-\bar{M}}{\sqrt{\frac{1}{len}\sum_{r=1}^{len}{\left(M_{r}-M\right)}^{2}}}}{len}$$

where *d* represents the lag value of autocorrelation, and *nlag* represents the maximum value of lag. In this study, the lag value *d* = 3 was selected; $M_{i}$ is the nucleotide properties at position *i*. $\bar{M^{\prime }}$ is calculated as follows:

(9) $$\bar{M\prime }=\frac{\sum_{i=1}^{N}M_{i}}{N}\;$$

Geary: Geary uses the attribute information of nucleotides to describe the sequence characteristics of miRNA. We calculate the miRNA sequence similarity, defined as follows:

(10) $$Geary\_\cos\_sim\left(i,\;j\right)=\frac{\sum_{j=1}^{n}\sum_{i=1}^{n}G{\left(d\right)}_{i}\times G{\left(d\right)}_{j}}{\sqrt{\sum_{i=1}^{n}{\left(G{\left(d\right)}_{i}\right)}^{2}}\times \sqrt{\sum_{j=1}^{n}{\left(G{\left(d\right)}_{j}\right)}^{2}}}$$

(11) $$Geary\_euc\_dist\left(i,j\right)=\sqrt{\overset{n}{\underset{\begin{array}{c} i=1\\ j=1 \end{array}}{\sum }}{\left(G{\left(d\right)}_{i}-G{\left(d\right)}_{j}\right)}^{2}}$$

(12) $$Geary\_pearson\left(i,j\right)=\frac{N\sum G{\left(d\right)}_{i}\times G{\left(d\right)}_{j}-\sum G{\left(d\right)}_{i}\sum G{\left(d\right)}_{j}}{\sqrt{N\sum G{\left(d\right)}_{i}{}^{2}-{\left(\sum G{\left(d\right)}_{i}\right)}^{2}}\sqrt{N\sum G{\left(d\right)}_{j}{}^{2}-{\left(\sum G{\left(d\right)}_{j}\right)}^{2}}}\;$$

where the Geary feature of miRNA are defined as follows:

(13) $$G\left(d\right)=\frac{\frac{1}{2\left(N-d\right)}\sum_{i=1}^{N-d}{\left(M_{i}-M_{i+d}\right)}^{2}}{\frac{1}{N-1}\sum_{i=1}^{N}{\left(M_{i}-\bar{M^{\prime }}\right)}^{2}},\;d=1,2,\ldots ,nlag$$

The meaning of physical and chemical indicators involving nucleotides, such as *d*, *M*, *M_i_*, and *nlag*, is the same as that in the Moran autocorrelation descriptor.

NMBroto: NMBroto is a normalized Moreau-Broto autocorrelation descriptor. The miRNA sequence is calculated similarity, defined as follows:

(14) $$NMBroto\_\cos\_sim\left(i,\;j\right)=\frac{\sum_{j=1}^{n}\sum_{i=1}^{n}NMB{\left(d\right)}_{i}\times NMB{\left(d\right)}_{j}}{\sqrt{\sum_{i=1}^{n}{\left(NMB{\left(d\right)}_{i}\right)}^{2}}\times \sqrt{\sum_{j=1}^{n}{\left(NMB{\left(d\right)}_{j}\right)}^{2}}}\;$$

(15) $$NMBroto\_euc\_dist\left(i,j\right)=\sqrt{\overset{n}{\underset{\begin{array}{c} i=1\\ j=1 \end{array}}{\sum }}{\left(NMB{\left(d\right)}_{i}-NMB{\left(d\right)}_{j}\right)}^{2}}\;$$

(16) $$NMBroto\_pearson\left(i,j\right)=\frac{N\sum NMB{\left(d\right)}_{i}\times NMB{\left(d\right)}_{j}-\sum NMB{\left(d\right)}_{i}\sum NMB{\left(d\right)}_{j}}{\sqrt{N\sum NMB{\left(d\right)}_{i}{}^{2}-{\left(\sum NMB{\left(d\right)}_{i}\right)}^{2}}\sqrt{N\sum NMB{\left(d\right)}_{j}{}^{2}-{\left(\sum NMB{\left(d\right)}_{j}\right)}^{2}}}$$

where the NMBroto feature of miRNA is defined as follows:

(17) $$NMB\left(d\right)=\frac{\sum_{i=1}^{N-d}M_{i}\times M_{i+d}}{N-d},\;d=1,2,\ldots ,nlag$$

The meaning of physical and chemical indicators involving nucleotides, such as *d*, *M*, *M_i_*, and *nlag*, is the same as that in the Moran autocorrelation descriptor.

CKSNAP: CKSNAP uses k-spaced nucleic acid pairs to describe the frequency of separating the current nucleic acid pair from any k nucleic acids. When k = 0, there are 16 pairs of nucleic acid pairs with 0 spacing (‘AA’, ‘AC’, ‘AG’, ‘AT’, ‘CA’, ‘CC’, ‘CG’, ‘CT’, ‘GA’, ‘GC’, ‘GG’, ‘GT’, ‘TA’, ‘TC’, ‘TG’, ‘TT’). The miRNA sequence was calculated similarity, defined as follows:

(18) $$CKSNAP\_\cos\_sim\left(i,\;j\right)=\frac{\sum_{j=1}^{n}\sum_{i=1}^{n}CK_{i}\times CK_{j}}{\sqrt{\sum_{i=1}^{n}{\left(CK_{i}\right)}^{2}}\times \sqrt{\sum_{j=1}^{n}{\left(CK_{j}\right)}^{2}}}\;$$

(19) $$CKSNAP\_euc\_dist\left(i,j\right)=\sqrt{\overset{n}{\underset{\begin{array}{c} i=1\\ j=1 \end{array}}{\sum }}{\left(CK_{i}-CK_{j}\right)}^{2}}\;$$

(20) $$CKSNAP\_pearson\left(i,j\right)=\frac{N\sum CK_{i}\times CK_{j}-\sum CK_{i}\sum CK_{j}}{\sqrt{N\sum CK_{i}{}^{2}-{\left(\sum CK_{i}\right)}^{2}}\sqrt{N\sum CK_{j}{}^{2}-{\left(\sum CK_{j}\right)}^{2}}}$$

where the CKSNAP features of miRNA are defined as follows:

(21) $$CK={\left(\frac{N_{AA}}{N_{total}},\;\frac{N_{AC}}{N_{total}},\;\frac{N_{AG}}{N_{total}},\ldots ,\;\frac{N_{TT}}{N_{total}},\right)}_{16}$$

Using five miRNA sequence features and three similarity calculation methods, 15 miRNA sequence similarity matrices could be obtained. In the following research, all sequence similarity matrices are called *MSSM*.

### 2.3. Disease Semantic Similarity

Disease semantic similarity can be calculated based on the medical subject heading (MeSH) descriptors, which are available at https://www.ncbi.nlm.nih.gov (accessed on 1 January 2021). The relationship between diseases can be represented as a directed acyclic graph (DAG) network according to disciplines or affiliations, where the nodes represent the MeSH descriptors of diseases, and the directed edges point from parent nodes to child nodes [35]. We adopted $DAG_{d_{i}}\left(d_{k}\right)$ to describe semantic contribution of disease *d_k_* to disease *d_i_*. On this basis, the semantic contribution is defined as follows:

(22) $$DAG_{d_{i}}\left(d_{k}\right)=\{\begin{array}{c} 1,\;if\;d_{k}=d_{i}\\ max\left\{\Delta *DAG_{d_{i}}\left({d^{\prime }}_{k}\right)|{d^{\prime }}_{k}\in children\;of\;d_{k}\right\},\;if\;d_{k}\neq d_{i} \end{array}$$

where ∆ is the semantic contribution attenuation facto; this was set to 0.5 according to a previous study [30]. The semantic contribution value will decrease with distance.

According to the semantic contribution value of disease nodes, the semantic value of disease *d_i_* was calculated as follows:

(23) $$DV\left(d_{i}\right)=\underset{d_{k}\in N\left(d_{i}\right)}{\sum }DAG_{d_{i}}\left(d_{k}\right)\;$$

The semantic similarity between disease *d_i_* and disease *d_k_* could be calculated by the nodes shared by the two disease DAG networks. It was defined as follows:

(24) SSM(di,dj)=∑(DAGdi(dt)+DAGdj(dt))dt∈N(di)∩N(dj) DV(di)+DV(dj) 

where the element *DSSM(d_i_, d_j_)* represents the disease semantic similarity between *d_i_* and *d_j_*.

### 2.4. Gaussian Interaction Profile Kernel Similarity for miRNAs and Diseases

In this study, the binary vector *IP(m_i_)* to denote the interaction profiles of miRNA *m_i_* was defined by calculating the correlation between *m_i_* and *m_j_*. The miRNA Gaussian interaction profile kernel similarity matrix (*MGSM*) could be calculated as follows:

(25) $$MGSM\left(m_{i},m_{j}\right)=\exp\left(-\sigma_{m}\|IP\left(m_{i}\right)-IP\left(m_{j}\right){\|}^{2}\right)$$

where σ*_m_* was applied for controlling the bandwidth of the kernel, and $\|IP\left(m_{i}\right)-IP\left(m_{j}\right){\|}^{2}$ was applied for calculating the euclidean distance of two eigenvectors. The Gaussian kernel bandwidth control parameter was calculated as follows:

(26) $$\sigma_{m}=\sigma \prime_{m}/\left(\frac{1}{nm}\overset{nm}{\underset{i=1}{\sum }}\|IP\left(m_{i}\right){\|}^{2}\right)$$

where *nm* represents the number of all miRNAs; $\sigma \prime_{m}$ was set to 1 according to a previous study [30]. Similarly, the Gaussian interaction profile kernel similarity for diseases *DGSM(d_i_,d_j_)* between disease *d_i_* and *d_j_* could be calculated as follows:

(27) $$DGSM\left(d_{i},d_{j}\right)=\exp\left(-\sigma_{d}\|IP\left(d_{i}\right)-IP\left(d_{j}\right){\|}^{2}\right)$$

(28) $$\sigma_{d}=\sigma \prime_{d}/\left(\frac{1}{nd}\overset{nd}{\underset{i=1}{\sum }}\|IP\left(d_{i}\right){\|}^{2}\right)$$

where *nd* represents the number of all diseases, $\sigma {’}_{d}$ was set to 1 according to a previous study [30].

### 2.5. Integrated Similarity for miRNAs and Diseases

There is a large number of sparse values in the miRNA sequence similarity and disease semantic similarity matrices. The final integrated similarity for miRNAs and diseases was obtained from miRNA sequence similarity, disease semantic similarity, Gaussian interaction profile kernel similarity for miRNAs, and Gaussian interaction profile kernel similarity for diseases. It was defined as follows:

(29) $$MSim\left(m_{i},m_{j}\right)=\{\begin{array}{l} MSSM\left(m_{i},m_{j}\right),\;if\;m_{i}\;and\;m_{j}\;have\;sequence\;similarity\\ MGSM\left(m_{i},m_{j}\right),\;otherwise \end{array}$$

(30) $$DSim\left(d_{i},d_{j}\right)=\{\begin{array}{l} DSSM\left(d_{i},d_{j}\right),\;if\;d_{i}\;and\;d_{j}\;have\;semantic\;similarity\;\\ DGSM\left(d_{i},d_{j}\right),\;otherwise \end{array}$$

### 2.6. Graph Auto-Encoder

**Figure 1:**
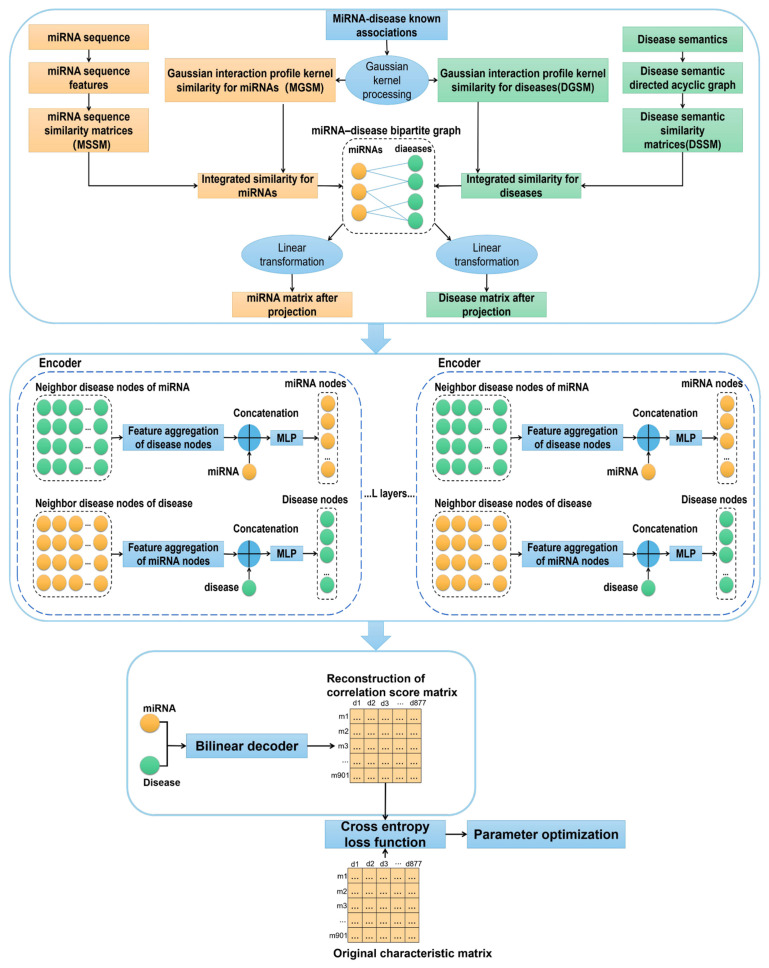
Technical route of miRNA disease correlation prediction model.

In the present study, the prediction of the potential miRNA–disease associations was mainly used to generate the low-dimensional embedding of node information through the encoder based on a graph neural network, so as to realize the identification of the correlation between miRNA and diseases by a bilinear decoder. Our model can be described in four steps, as shown in Figure 1.

First, we constructed a bipartite graph including 877 disease nodes and 901 miRNA nodes. For integrated similarity for miRNA, the similarity between miRNA *m_i_* and miRNA *m*_1_, *m*_2_, …, *m*_901_ could be expressed as follows:

(31) $$F_{m}\left(i\right)=\left(u_{1},u_{2},u_{3},\ldots ,u_{901}\right)$$

where *u*_1,_ *u*_2_*, u*_3_*, …, u*_901_ is the integrated similarity between miRNA *m(i)* and miRNA *m*_1_, *m*_2_, …, *m*_901_. Similarly, the integrated similarity between disease *d_i_* and disease *d*_1_, *d*_2_, …, *d*_901_ could be expressed as follows:

(32) $$F_{d}\left(i\right)=\left(v_{1},v_{2},v_{3},\ldots ,v_{877}\right)$$

The vectors in *F_m_* (miRNA) and *F_d_* (disease) were embedded into miRNA nodes and disease nodes in the miRNA–disease bipartite graph. In this study, 16,427 known association pairs verified by experiments were regarded as positive samples of input. In order to balance the positive and negative samples, 16,427 negative samples were randomly selected from the unknown associations in the subsequent experiments. In order to project miRNA and disease feature vectors to the same vector space, we designed a linear transformation matrix. The projection process of miRNA nodes can be described as follows:

(33) $$H_{m}=W_{\emptyset_{m}}\cdot F_{m}$$

where *F_m_* is the original characteristic matrix of miRNA, $W_{\emptyset_{m}}$ is to realize the linear transformation matrix of miRNA projection process, and *H_m_* is the feature matrix of miRNA projected into the new feature space. Similarly, the projection process of disease nodes can be described as follows:

(34) $$H_{d}=W_{\emptyset_{d}}\cdot F_{d}$$

where *F_d_*, $W_{\emptyset_{m}}$, and *H_d_* are as described above.

Second, we used the aggregator function of the auto-encoder to integrate the feature representation of the node and its neighbor nodes, update the node with a multi-layer perceptron, and embed the aggregated features into the original features of the node. The aggregator function *sum(·)* was used to realize the feature aggregation of its neighbor nodes, as shown in Equations (35) and (36), where $sum\left(H_{d}\left(1\right),H_{d}\left(2\right),\ldots \right)$ represents the aggregation process of the disease nodes *d_j_*., which are the direct neighbors of the miRNA nodes *m_i_*; *Dm_i_* is the degree value of node *m_i_*. In order to prevent numerical instability, $H_{m}^{s}\left(i\right)$ was used for normalization. It is the aggregation feature of the current miRNA node *m_i_*.

(35) $$H_{m}^{s}\left(i\right)=\frac{1}{D_{m_{i}}}sum\left(H_{d}\left(1\right),H_{d}\left(2\right),\ldots \right)$$

(36) $$D_{m_{i}}=\left|\left\{d_{j}|\exists e_{ij}\in E\;or\;e_{ji}\in E\right\}\right|$$

After the aggregation features of all miRNA nodes were obtained through the aggregator function, the features of all nodes were embedded and connected through the multi-layer perceptron superimposed by the L-layer, and the final embedding of nodes was generated. We used a Leaky Rectified Linear Unit (LeakyReLU) function as the activation function of the multilayer perceptron to avoid the phenomenon of neuron “death” when the input was negative. It is defined as follows:

(37) $${H^{\prime }}_{m}\left(i\right)=LeakyReLU\left(f\left(H_{m}\left(i\right)⊕H_{m}^{s}\left(i\right)\right)\right)$$

where $H_{m}\left(i\right)$ is the original feature of node *m_i_*, $H_{m}^{s}\left(i\right)$ is the aggregation feature of node *m_i_*, ${H^{\prime }}_{m}\left(i\right)$ is the updated node feature, and *f*(·) is the single-layer multi-layer perceptron function. Similarly, we calculated the aggregation feature of the current disease node *d_j_*, the degree value of node *d_j_*, and the updated node feature of disease as in Equations (38)–(40).

(38) $$H_{d}^{s}\left(j\right)=\frac{1}{D_{d}\left(j\right)}sum\left(H_{m}\left(1\right),H_{m}\left(2\right),\ldots \right)$$

(39) $$D_{d}\left(j\right)=\left|\left\{m_{i}|\exists e_{ij}\in E\;or\;e_{ji}\in E\right\}\right|$$

(40) $${H^{\prime }}_{d}\left(j\right)=LeakyReLU\left(f\left(H_{d}\left(j\right)⊕H_{d}^{s}\left(j\right)\right)\right)$$

A multilayer overlay network could enhance the feature embedding of neighbor nodes and preserve the topology of graph data, so as to enhance the expression ability of features.

Third, considering that the number of unknown associations between miRNA and disease is much larger than the known associations, we adopted a sigmoid function as the activation function of the decoder to predict the association score between miRNA and disease. It is defined as follows:

(41) $$\hat{A}\left(i,j\right)=sigmoid\left(H_{d}^{L}\left(j\right)*Q{\left(H_{m}^{L}\left(i\right)\right)}^{T}\right)$$

where *Q* is the E-dimensional trainable parameter matrix, $H_{d}^{L}\left(j\right)$ is the feature embedding of L-layer disease node *d_j_*, $H_{m}^{L}\left(i\right)$ is the feature embedding of L-layer miRNA node *m_i_*, and $\hat{A}\left(i,j\right)$ is the reconstructed association score matrix.

Finally, we used the deviation between the prediction score matrix and the original characteristic matrix and used the cross-entropy loss function and back propagation algorithm to optimize the model to obtain the best model parameters. It is defined as follows:

(42) $$LOSS=-\underset{i,j\in y\cup y-}{\sum }\left(A\left(i,j\right)*log\hat{A}\left(i,j\right)+\left(1-A\left(i,j\right)\right)*\log\left(1-\hat{A}\left(i,j\right)\right)\right)$$

where A(i,j) is the original characteristic matrix, and $\hat{A}\left(i,j\right)$ is the reconstructed prediction correlation score matrix. Due to the small proportion of known associations in the sample data, cost-sensitive learning was used to improve the weight of positive sample loss, so as to improve the prediction accuracy. Furthermore, *y* and *y*- are the set of positive samples and the set of negative samples in the incidence matrix, respectively.

### 2.7. Model Evaluation

Five-fold cross validation was selected to evaluate the performance of the model. We divided the miRNA–disease associations into five sets, set one as the test set and the other four as the training set, and received five prediction results. Accordingly, the higher the score between miRNA and disease, the higher the possibility of a potential association between them. In the present study, we adopted four common evaluating indicators to evaluate the performance of the models: Accuracy (Acc), Precision (Prec), Recall, and F1-score. Meanwhile, we plotted the receiver operating characteristic curves to intuitively display the performance of our model and utilized the AUC to comprehensively evaluate model performance.

## 3. Results

### 3.1. Performance Evaluation of Graph Neural Network Prediction Model Based on Single Features

**Figure 2:**
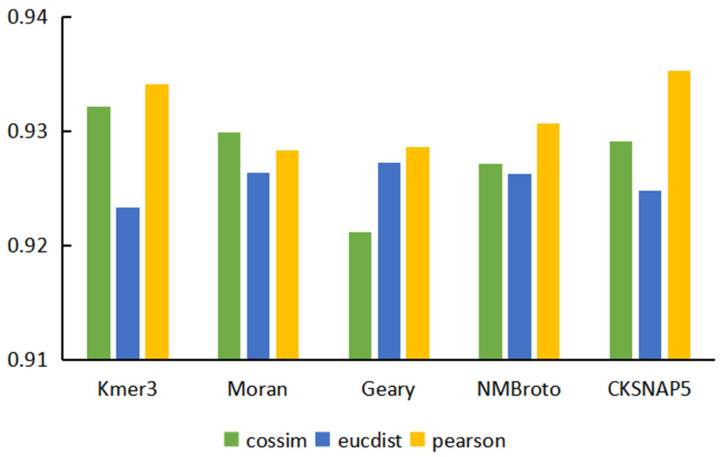
AUC of models based on different features and different similarity calculation methods.

Based on five sequence features, we used three different similarity calculation methods to obtain 15 different miRNA sequence similarity matrices and constructed 15 prediction models. The performance comparison of the prediction models based on different characteristics and similarity calculation methods is shown in Figure 2. The overall prediction performance of the model for calculating sequence feature similarity based on the Pearson correlation coefficient was better than the other two similarity calculation methods. This type of model obtained the optimal values in AUC, ACC, precision, and F1-score, and had obvious advantages over the other two similarity algorithms. Comparing the performance of the model based on five features, we found that the performance of the model based on K-mer and CKSNAP was better than the other models. Considering that AUC could more comprehensively evaluate model performance, the model based on CKSNAP sequence features and the Pearson similarity calculation method obtained the highest AUC value among the 15 models. In addition, this model was also better than other models in ACC, precision, and F1 scores.

### 3.2. Performance Evaluation of Graph Neural Network Prediction Model Based on Combined Features

**Figure 3:**
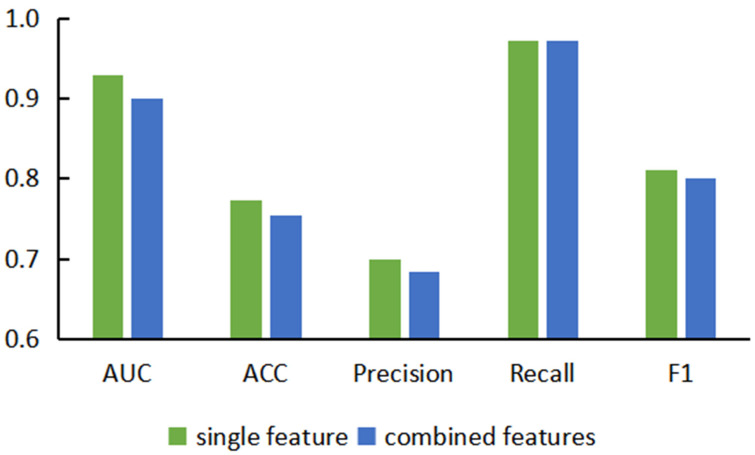
Performance comparison diagram of combined feature-based models and single feature-based models.

Based on the comparison and analysis of model performance based on single features, we proposed prediction models based on combined features, hoping to enhance the expression ability of nodes by embedding multiple features on a single node. Here, we combined the five features in pairs to obtain 10 combined features and used different similarity calculation methods to build 30 different prediction models based on these combined features. In order to compare the performance of the two types of models more intuitively, we classified these two types of models and calculated their average scores on each evaluation indicator and drew the prediction performance based on single-feature and double-feature models, as shown in Figure 3. We noticed that the evaluation indicators of models based on combined features were lower than those of the models based on single features. We speculate that this is because the dimensions of the combined features were too high. Embedding more feature information into a single node also introduced too much noise and redundant information, which led to model instability in the subsequent experimental process, resulting in a decline in prediction performance.

**Figure 4:**
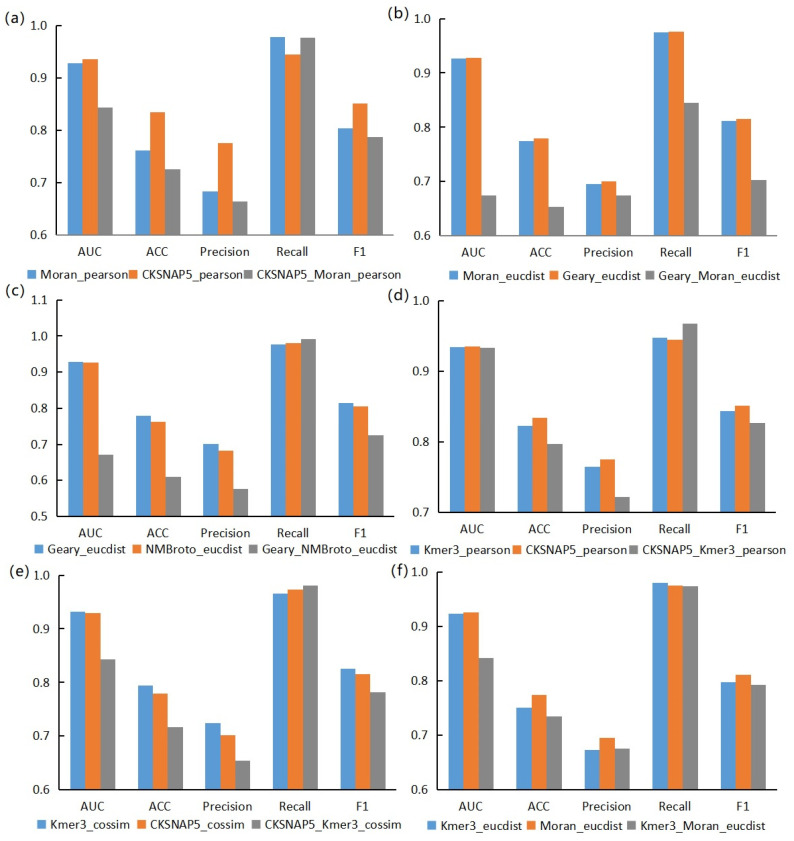
Example diagram of dependence of dual feature models on single feature models. (**a**) is a comparison between the Pearson correlation coefficient model based on Moran feature (blue), the Pearson correlation coefficient model based on CKSNAP feature (dark orange) and the Pearson correlation coefficient model based on Moran+CKSNAP combined feature (gray). (**b**) is a comparison between the Euclidean distance model based on Moran feature (blue), the Euclidean distance model based on Geary feature (dark orange) and the Euclidean distance model based on Moran+Geary combined feature (gray). (**c**) is a comparison between the Euclidean distance model based on Geary feature (blue), the Euclidean distance model based on NMBroto feature (dark orange) and the Euclidean distance model based on Geary+NMBroto combined feature (gray). (**d**) is a comparison between the Pearson correlation coefficient model based on Kmer feature (blue), the Pearson correlation coefficient model based on CKSNAP feature (dark orange) and the Pearson correlation coefficient model based on Kmer+CKSNAP combined feature (gray). (**e**) is a comparison between the cosine similarity model based on Kmer feature (blue) and the cosine similarity model based on CKSNAP feature (yellow) and the cosine similarity model based on Kmer+CKSNAP combined feature (gray). (**f**) is a comparison between the Euclidean distance model based on Kmer feature (blue), the Euclidean distance model based on Moran feature (dark orange) and the Euclidean distance model based on Kmer+Moran combined feature (gray).

In addition, we also found that the score of the models based on combined features depended on the score of the models based on single features. Here, we selected six groups of models for display, as shown in Figure 4. It can be seen that the models based on single features were better than the models based on combined features in AUC, Acc, Prec, and F1-score. In Figure 4, the gray columns are mostly located below the other two columns. The recall rate of models based on combined features in individual groups is higher. The reason for this is that the combined features enrich the information range, which leads to the improvement of recall rate. Combined features could enrich the information contained in a single node, which would make the coverage of information and the construction of relationship structure in the whole network more comprehensive, resulting in the improvement of Recall. However, due to the high dimensions of the feature vector and too much noise information on a single node, other evaluation indicators would decline.

Based on this, the subsequent experiments focused on performance optimization based on single-feature models. In a preliminary study, we set project dimension to E = 256 and set encoder layers to L = 2.

### 3.3. Effects of Projection Dimension and Encoder Layers on Model Performance

**Figure 5:**
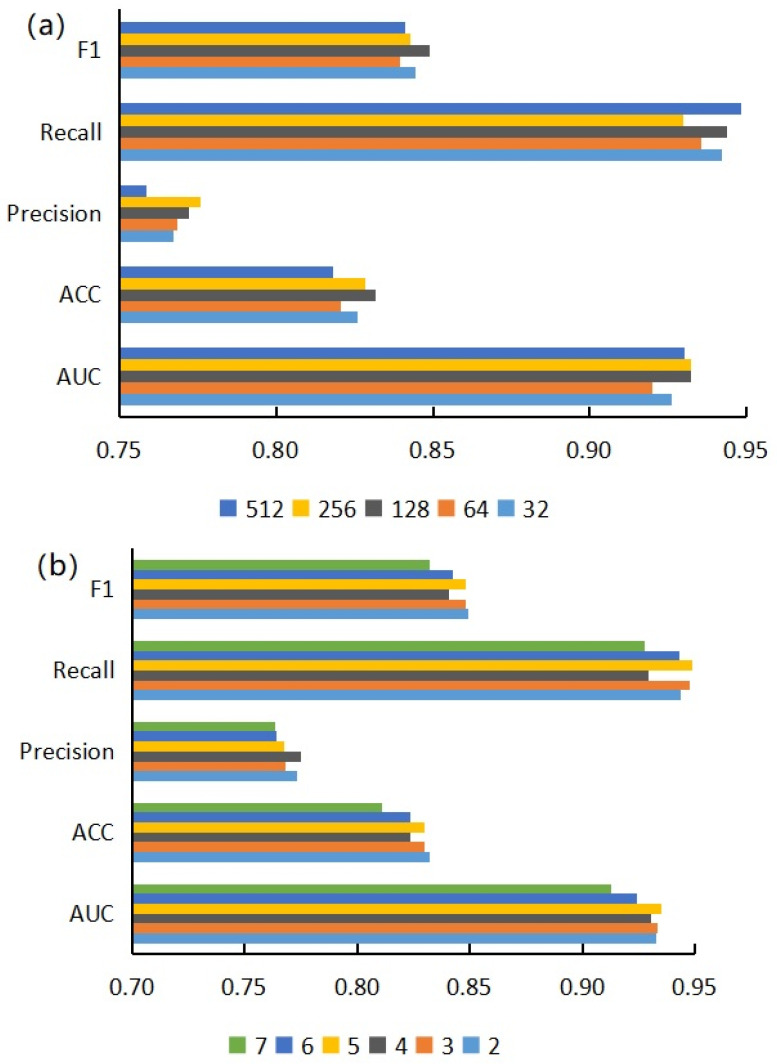
(**a**) Effects of different projection dimensions on model performance; (**b**) influence of different encoder layers on model performance.

Here, we used the model based on CKSNAP features and the Pearson similarity calculation method to study the influence of projection dimension and encoder layers on prediction performance, changing the number of projection dimensions and encoder layers to obtain the scores of different models on five evaluation indicators. Different projection dimensions will affect the expression ability of nodes. Changing the number of encoder layers can adjust the learning degree of neural networks to node feature information. As can be seen from Figure 5a, with an increase in projection dimensions, the recall rate of the model fluctuated greatly, and the change in F1 score was not obvious. The accuracy score increased significantly with the increase of projection dimensions but dropped sharply when the projection dimension, E, was greater than 256. According to Figure 5b, with an increase in encoder layers, the evaluation indexes of the model showed a downward trend as a whole; especially after L > 6, the indexes of the model declined sharply.

### 3.4. Performance Evaluation and Comparative Analysis of Related Models

**Figure 6:**
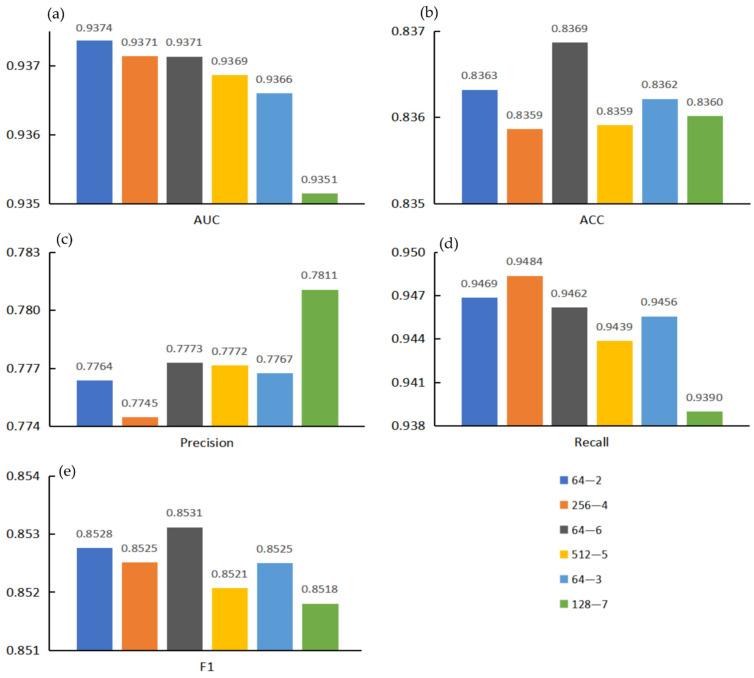
The top five evaluation indicators were marked and summarized to obtain two or more models. (**a**) AUC value comparison; (**b**) ACC value comparison; (**c**) Precision value comparison; (**d**) Recall value comparison; (**e**) F1 value comparison.

**Figure 7:**
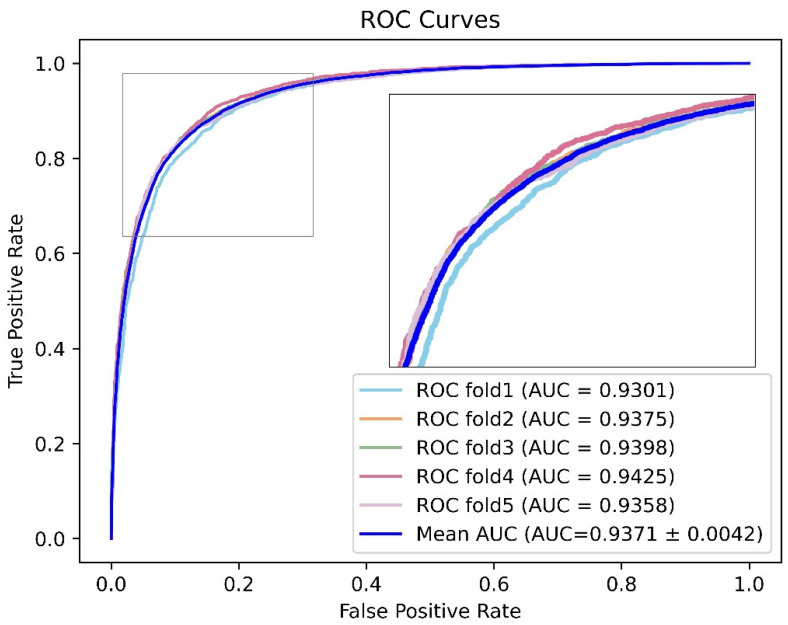
The model with project dimension E = 64 and encoder layers L = 6 had better performance.

Here, we marked the top five models in each evaluation indicator and screened out the models with two or more markers to obtain the results shown in Figure 6. Considering that Acc was greatly affected by the proportion of positive samples in the sample set, Acc was not taken as the primary evaluation indicator. Precision and recall provide a single view of the performance, whereas F1 score provides a more comprehensive view of the performance and therefore was used for model evaluation. Therefore, considering AUC and F1 score, we noted that the model with project dimensions E = 64 and encoder layer L = 6 had better performance in all aspects. The receiver operating characteristic curve for the 5-fold cross validation experiment is shown in Figure 7. At the same time, the ROC curve of each fold can be seen in Figure S1.

In order to further evaluate the performance of this model, we compared it with six related models (PBMDA [36], LLCMDA [37], EDTMDA [38], GBDTLR [20], MCLPMDA [39], GAEMDA [40]) for comprehensive evaluation. Considering that different studies used different evaluation indicators, only the AUC value that could comprehensively evaluate the performance of the model was selected for comparative analysis. Our study selected the optimal AUC recorded in each paper for comparison, as shown in Table 1. Among the seven models, our model obtained the highest AUC value, which was 0.15% higher than the AUC value of the model with the second highest, GAEMDA.

### 3.5. Case Studies

In recent years, more and more research has shown that the mutation or abnormal expression of miRNA causes many human diseases [41,42]. In order to further evaluate the performance of our prediction algorithm, three neoplasm diseases were selected for independent case studies—lung neoplasm, esophageal neoplasm, and kidney neoplasm—as it done in the reported method using the same dataset. We deleted the specific diseases of the case study from the training samples to remove bias from the experiments. We used the remaining miRNAs and diseases to construct test samples and ranked them according to the prediction scores. We compared the top 50 prediction results with the dbDEMC databases to obtain the prediction results.

Lung neoplasm is a malignant neoplasm disease occurring in lung parenchyma and stroma [43]. Lung cancer is one of the diseases with the fastest growth rate of morbidity and mortality. It has become the most common cause of death in malignant tumors; the prediction results of our model for miRNA related to lung neoplasm are shown in Table 2. It can be seen that 47 of the top 50 miRNAs could be verified in the dbDEMC database. Esophageal neoplasm is a malignant neoplasm disease that occurs in esophageal epithelial tissue [44,45,46]. Approximately 300,000 people die of esophageal cancer every year in the world. China is one of the high incidence areas of esophageal cancer in the world; the prediction results of our model for miRNA related to esophageal neoplasm are shown in Table 3. It can be seen that 47 of the top 50 miRNAs could be verified in the dbDEMC database. Kidney neoplasm is a common neoplasm disease in the urinary system. The pathological structure of kidney neoplasm is complex, and the cause of disease is variable [47]. Finding a new entry point for diagnosis is of great significance for the timely targeting of patients. The prediction results of our model are shown in Table 4. It can be seen that 37 of the top 50 miRNAs could be verified in the dbDEMC database.

## 4. Conclusions

In the present study, we used a variety of miRNA sequence features to better retain the sequence similarity information between miRNAs and used a disease–miRNA bipartite graph to mine for potential deeper association information between miRNAs and diseases. Under a 5-fold cross validation, the experimental results showed that the prediction performance of the prediction algorithm based on combined features was not as good as that based on single features, and there was a dependency between the prediction score based on combined features and the prediction model based on corresponding single features. We used this model for case study verification, and the prediction results of three specific tumors also achieved a good hit rate. Therefore, our research is helpful for researchers to quickly and effectively study the relationship between miRNAs and diseases and plays a guiding role in research. It can save time and the cost of wet experiments to find disease-relevant miRNAs. This method can be used to predict the miRNA associated with a disease, and then perform wet experiment verification. Alternatively, there are results from wet experiments, and the method could be used to provide a confidence to refer to in experimental results. However, the analysis and discussion described in this paper is only a small part of the research on the correlation between miRNAs and disease, and there are more questions to be explored. For example, it could be useful to embed more biological information in the structural association between disease and miRNA. For example, when calculating the sequence similarity of miRNAs, we can consider introducing the functional similarity of miRNAs, the MISIM network, and the correlation information between miRNAs and proteins. Further work can focus on finding methods that can obtain deep-seated network structure information without affecting the prediction performance of the model. Finally, considering that the regulatory mechanisms of miRNA in many complex diseases also play an important role in miRNA–disease associations, the analysis of model performance combined with physiological influencing factors is expected to improve future experiments.

## Figures and Tables

**Table 1:** Comparative analysis of our model and related models.

Model	AUC (%)
PBMDA	91.72
LLCMDA	91.90
EDTMDA	91.92
GBDTLR	92.74
MCLPMDA	93.20
GAEMDA	93.56
Our Model	93.71

**Table 2:** Lung neoplasm associated miRNA Top 50 predicted by hmdd v3.2.

miRNA	dbDEMC	miRNA	dbDEMC
hsa-mir-586	Confirmed	hsa-mir-329-5p	Confirmed
hsa-mir-208b-5p	Confirmed	hsa-mir-1264	Confirmed
hsa-mir-376b-5p	Confirmed	hsa-mir-618	Confirmed
hsa-mir-3613-5p	Confirmed	hsa-mir-599	Confirmed
hsa-mir-4775	Confirmed	hsa-mir-517c-3p	Unconfirmed
hsa-mir-544a	Confirmed	hsa-mir-384	Confirmed
hsa-mir-450a-5p	Confirmed	hsa-mir-581	Confirmed
hsa-mir-376c-5p	Confirmed	hsa-mir-578	Confirmed
hsa-mir-376a-5p	Confirmed	hsa-mir-19b-2-5p	Confirmed
hsa-mir-190a-5p	Confirmed	hsa-mir-552-5p	Confirmed
hsa-mir-875-5p	Confirmed	hsa-mir-5590-5p	Confirmed
hsa-mir-3682-5p	Confirmed	hsa-mir-450a-1-3p	Confirmed
hsa-mir-302f	Confirmed	hsa-mir-454-5p	Confirmed
hsa-mir-5586-5p	Confirmed	hsa-mir-942-5p	Confirmed
hsa-mir-450b-5p	Confirmed	hsa-mir-548l	Confirmed
hsa-mir-576-5p	Confirmed	hsa-mir-548k	Confirmed
hsa-mir-4295	Confirmed	hsa-mir-1185-5p	Confirmed
hsa-mir-1282	Confirmed	hsa-mir-548am-5p	Confirmed
hsa-mir-5009-5p	Confirmed	hsa-mir-613	Confirmed
hsa-mir-655-5p	Confirmed	hsa-mir-1248	Confirmed
hsa-mir-16-2-3p	Confirmed	hsa-mir-544b	Confirmed
hsa-mir-548d-5p	Confirmed	hsa-mir-3913-5p	Confirmed
hsa-mir-1179	Confirmed	hsa-mir-548c-5p	Confirmed
hsa-mir-876-5p	Confirmed	hsa-mir-570-5p	Unconfirmed
hsa-mir-1206	Unconfirmed	hsa-mir-651-5p	Confirmed

**Table 3:** Esophageal neoplasm associated miRNA Top 50 predicted by hmdd v3.2.

miRNA	dbDEMC	miRNA	dbDEMC
hsa-mir-1179	Confirmed	hsa-mir-450b-5p	Confirmed
hsa-mir-1206	Confirmed	hsa-mir-4775	Confirmed
hsa-mir-1264	Confirmed	hsa-mir-493-5p	Confirmed
hsa-mir-1282	Confirmed	hsa-mir-495-5p	Confirmed
hsa-mir-135a-5p	Confirmed	hsa-mir-5009-5p	Confirmed
hsa-mir-136-5p	Confirmed	hsa-mir-517c-3p	Confirmed
hsa-mir-16-2-3p	Confirmed	hsa-mir-544a	Confirmed
hsa-mir-190a-5p	Confirmed	hsa-mir-545-5p	Confirmed
hsa-mir-196a-5p	Confirmed	hsa-mir-548d-5p	Confirmed
hsa-mir-199b-5p	Confirmed	hsa-mir-552-5p	Unconfirmed
hsa-mir-19b-2-5p	Confirmed	hsa-mir-5586-5p	Confirmed
hsa-mir-202-5p	Confirmed	hsa-mir-5590-5p	Confirmed
hsa-mir-208b-5p	Confirmed	hsa-mir-576-5p	Confirmed
hsa-mir-29a-5p	Confirmed	hsa-mir-578	Confirmed
hsa-mir-329-5p	Unconfirmed	hsa-mir-581	Confirmed
hsa-mir-3613-5p	Confirmed	hsa-mir-586	Confirmed
hsa-mir-3682-5p	Confirmed	hsa-mir-599	Confirmed
hsa-mir-376a-2-5p	Confirmed	hsa-mir-618	Confirmed
hsa-mir-376a-5p	Confirmed	hsa-mir-655-5p	Confirmed
hsa-mir-376c-5p	Confirmed	hsa-mir-7-5p	Confirmed
hsa-mir-384	Confirmed	hsa-mir-875-5p	Confirmed
hsa-mir-4295	Confirmed	hsa-mir-876-5p	Confirmed
hsa-mir-4423-5p	Confirmed	hsa-mir-95-5p	Confirmed
hsa-mir-450a-1-3p	Unconfirmed	hsa-mir-9-5p	Confirmed
hsa-mir-450a-5p	Confirmed	hsa-mir-29b-1-5p	Confirmed

**Table 4:** Kidney neoplasm associated miRNA Top 50 predicted by hmdd v3.2.

miRNA	dbDEMC	miRNA	dbDEMC
hsa-mir-105-5p	Confirmed	hsa-mir-449a	Confirmed
hsa-mir-1179	Confirmed	hsa-mir-449c-5p	Confirmed
hsa-mir-1204	Confirmed	hsa-mir-4775	Confirmed
hsa-mir-1244	Confirmed	hsa-mir-4795-5p	Unconfirmed
hsa-mir-1264	Confirmed	hsa-mir-517c-3p	Confirmed
hsa-mir-1267	Confirmed	hsa-mir-5193	Unconfirmed
hsa-mir-1282	Confirmed	hsa-mir-520h	Unconfirmed
hsa-mir-1284	Confirmed	hsa-mir-543	Confirmed
hsa-mir-1322	Confirmed	hsa-mir-548c-5p	Unconfirmed
hsa-mir-135b-5p	Confirmed	hsa-mir-5692b	Unconfirmed
hsa-mir-136-5p	Confirmed	hsa-mir-576-5p	Confirmed
hsa-mir-147b-5p	Unconfirmed	hsa-mir-577	Confirmed
hsa-mir-149-5p	Confirmed	hsa-mir-586	Confirmed
hsa-mir-18b-5p	Confirmed	hsa-mir-606	Confirmed
hsa-mir-202-5p	Confirmed	hsa-mir-616-5p	Confirmed
hsa-mir-212-5p	Confirmed	hsa-mir-626	Unconfirmed
hsa-mir-23c	Confirmed	hsa-mir-633	Confirmed
hsa-mir-3120-5p	Unconfirmed	hsa-mir-644a	Unconfirmed
hsa-mir-3149	Confirmed	hsa-mir-645	Confirmed
hsa-mir-32-5p	Unconfirmed	hsa-mir-764	Unconfirmed
hsa-mir-340-5p	Confirmed	hsa-mir-889-5p	Unconfirmed
hsa-mir-3662	Confirmed	hsa-mir-934	Confirmed
hsa-mir-3682-5p	Unconfirmed	hsa-mir-942-5p	Confirmed
hsa-mir-4295	Confirmed	hsa-mir-943	Confirmed
hsa-mir-4443	Confirmed	hsa-mir-944	Confirmed

## Data Availability

Not applicable.

## Supplementary Materials

The following supporting information can be downloaded at: https://www.mdpi.com/article/10.3390/genes13101759/s1, Figure S1. Receiver operating characteristic curve for each fold experiment. The source code can be downloaded from http://public.aibiochem.net/DNA_RNA/Genes_Human-miRNA-disease-Associations/code.zip (accessed on 1 January 2022).
