# Supplementary figures and images for: Using Sequence Similarity Based on CKSNP Features and a Graph Neural Network Model to Identify miRNA–Disease Associations
Source: Genes (Basel). 2022 Sep 28;13(10):1759. doi: 10.3390/genes13101759 (PMC9602123; doi:10.3390/genes13101759)

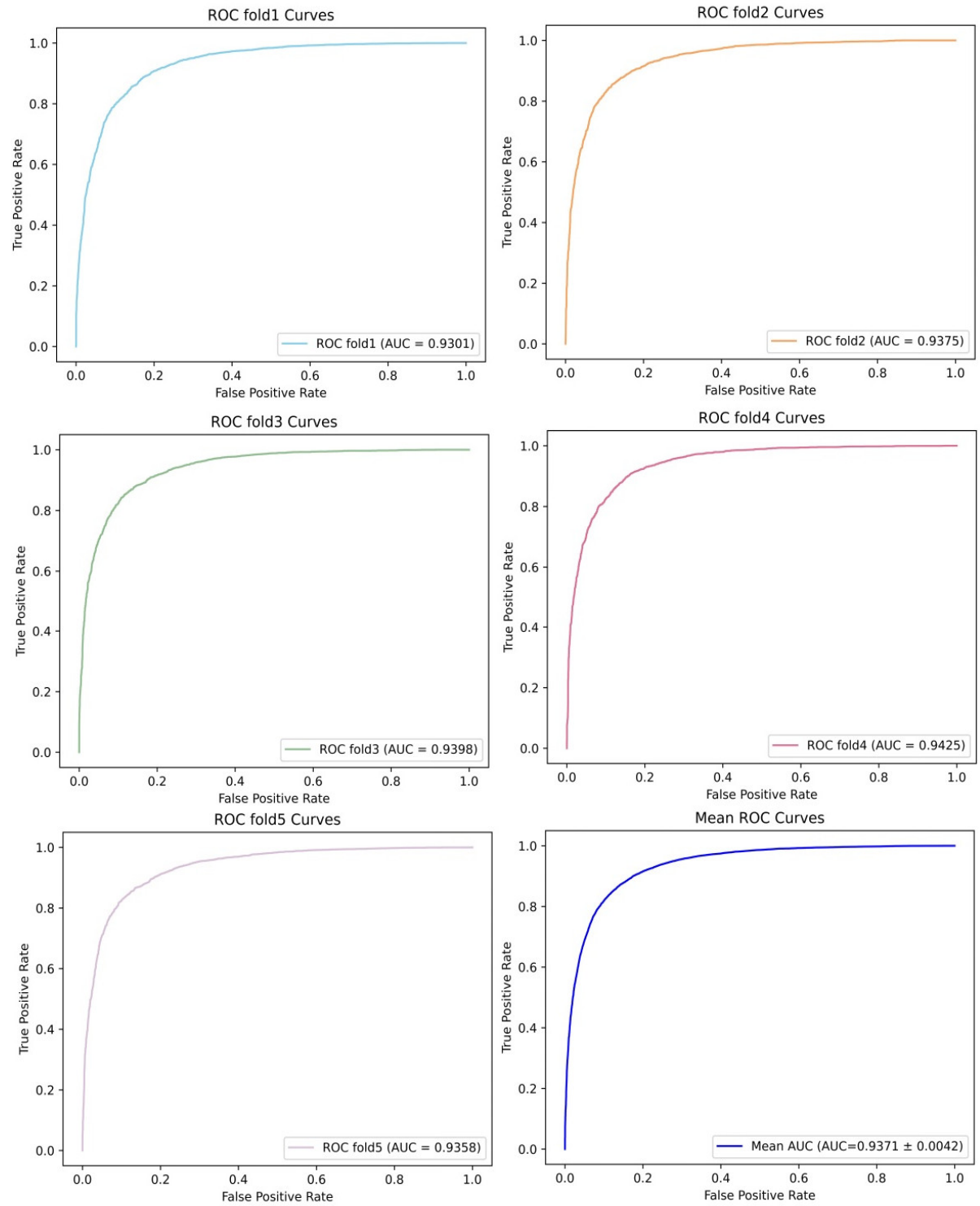

**Figure S1.** Receiver operating characteristic curve for each fold experiment.

Supplement: Supplementary file 1 [file genes-13-01759-s001.zip › genes-1876765-supplementary.pdf]
